# Time-of-day specificity of anticancer drugs may be mediated by circadian regulation of the cell cycle

**DOI:** 10.1126/sciadv.abd2645

**Published:** 2021-02-12

**Authors:** Yool Lee, Shi Yi Fong, Joy Shon, Shirley L. Zhang, Rebekah Brooks, Nicholas F. Lahens, Dechun Chen, Chi Van Dang, Jeffrey M. Field, Amita Sehgal

**Affiliations:** 1 Howard Hughes Medical Institute, Chronobiology and Sleep Institute (CSI), Perelman School of Medicine, University of Pennsylvania, Philadelphia, PA 19104, USA.; 2 Cell and Molecular Biology Graduate Group (CAMB), University of Pennsylvania, Philadelphia, PA 19104, USA.; 3 The Wistar Institute, Philadelphia, PA 19104, USA.; 4 Institute for Translational Medicine and Therapeutics, Perelman School of Medicine, University of Pennsylvania, Philadelphia, PA 19104, USA.; 5 Ludwig Institute for Cancer Research, New York, NY 10017, USA.; 6 Department of Systems Pharmacology and Translational Therapeutics, Perelman School of Medicine, University of Pennsylvania, Philadelphia, PA 19104, USA.

## Abstract

Circadian rhythms are an integral part of physiology, underscoring their relevance for the treatment of disease. We conducted cell-based high-throughput screening to investigate time-of-day influences on the activity of known antitumor agents and found that many compounds exhibit daily rhythms of cytotoxicity concomitant with previously reported oscillations of target genes. Rhythmic action of HSP90 inhibitors was mediated by specific isoforms of HSP90, genetic perturbation of which affected the cell cycle. Furthermore, clock mutants affected the cell cycle in parallel with abrogating rhythms of cytotoxicity, and pharmacological inhibition of the cell cycle also eliminated rhythmic drug effects. An HSP90 inhibitor reduced growth rate of a mouse melanoma in a time-of-day–specific manner, but efficacy was impaired in clock-deficient tumors. These results provide a powerful rationale for appropriate daily timing of anticancer drugs and suggest circadian regulation of the cell cycle within the tumor as an underlying mechanism.

## Teaser

Circadian regulation of tumors provides a basis for chemotherapy at specific times of day.

## INTRODUCTION

Circadian clocks generate 24-hour rhythms of physiology and behavior in nearly all living organisms. These conserved timing systems are thought to confer the host species with survival benefits by enabling adaptation of physiology with cyclic environmental changes (e.g., light, food availability, and predation). In mammals, endogenous circadian clocks occur throughout the body, from the master clock in the brain suprachiasmatic nucleus (SCN) down to single neurons and fibroblasts, maintaining daily rhythms of sleep/wake, feeding/fasting neuronal, endocrine, immune, and metabolic functions (*1*, *2*). At the molecular level, cell-autonomous rhythms are driven primarily by an autoregulatory feedback loop in which the BMAL1 and CLOCK transcription factor complex cyclically activates transcription of its own repressors, Period (PER)/Cryptochrome (CRY). The core oscillator is complemented by a second loop in which periodic expression of BMAL1 is maintained by the REV-ERBα/β repressor and RORα/β activator proteins (*3*). With additional levels of posttranscriptional and posttranslational processing, the molecular clockwork coordinates temporal programs via multiple clock-output genes (*4*).

The internal circadian system also influences organismal responses to exogenous cues such as food and drug intake, exercise, viral infection, and vaccination (*5*–*7*). Global circadian expression profiling studies reveal that most physiological pathways are characterized by rhythmically expressed genes, which include therapeutic targets (*8*, *9*). Thus, the case has been made for exploiting circadian rhythms to improve drug therapies for various diseases including cancer (*10*, *11*).

The nature of the link between clocks and cancer remains a topic of debate. For instance, circadian rhythms are abrogated in many cancers (*12*–*14*), and loss of circadian regulation is even implicated in the onset/progression of cancer, with genetic and environmental alterations of circadian rhythms increasing susceptibility to cancer pathogenesis (*15*, *16*). On the other hand, several cancers harbor a functional circadian clock that drives daily oscillations of gene expression (*17*, *18*), but little is known about the contribution of these clocks to tumor growth or treatment. Although chemotherapy timed to the host’s circadian rhythms can be more effective, the site and nature of circadian regulation required remain unclear (*19*, *20*). In general, the lack of experimental models and mechanistic evidence for circadian-modulated drug activity has limited the scope of timed chemotherapy treatment for cancer (chronochemotherapy) (*21*).

To determine the extent to which circadian time matters for chemotherapeutic reagents, we developed a cell-based assay and screened known anticancer compounds. In high- to low-throughput analysis using control or clock-deficient tumor cells, we found that several anticancer drugs exhibit rhythmic antiproliferative activity under the control of the circadian clock. Focusing on a subset of heat shock protein 90 (HSP90) inhibitors identified in the original assay, we show that the rhythmic action of these drugs relies upon specific cyclically expressed isoforms of HSP90. Isoforms of HSP90 required for rhythmic drug responses also affect the cell cycle, suggesting that rhythms of drug response rely upon a synchronized cell cycle. 17-DMAG, a potent HSP90 inhibitor, displays time-of-day–specific action on melanoma tumors in mice but is less effective on clock mutant tumors even in a wild-type host, underscoring the importance of circadian regulation within the tumor. This study provides a molecular rationale and mechanistic evidence for timed application of anticancer medicine and treatment.

## RESULTS

### Screen of anticancer drugs for time-of-day–specific effects

We recently reported a 24-hour rhythm in the antiproliferation activity of a cell cycle inhibitor (*15*). To determine the extent to which other anticancer drugs have time-of-day specificity, we conducted a high-throughput screen (HTS) using human U2OS cells. The screen used a 126-drug panel that includes a comprehensive set of anticancer compounds targeting multiple signaling pathways such as those involved in cell survival (Raf/Ras and Fgfr/Pdgfr/Vegfr), cell cycle control (Cdk, Plk, Aurk, Wee1, CHK1, and ATM), autophagy (mTor/PI3K/Akt), and apoptosis (p53/bcl2/Bimp) (table S1).

We first synchronized circadian rhythms in U2OS cells by treating with 100 nM dexamethasone (dex). Drugs were delivered at different circadian phases relative to the time of synchronization with dex, and each drug was tested at eight different serial dilutions. After 72 hours of incubation with drugs, we measured cell viability by determining the adenosine 5′-triphosphate (ATP) content in each well with ATPlite (fig. S1). This was done for all 126 drugs, so each well differed from others by one or more factors: time of treatment relative to dex stimulation, specific drug used, and drug concentration. We determined the efficacy of each drug by calculating its median inhibitory concentration (IC_50_) value from the eight serial dilutions using a nonlinear curve fitting algorithm in GraphPad Prism software (fig. S1).

**Fig. 1:**
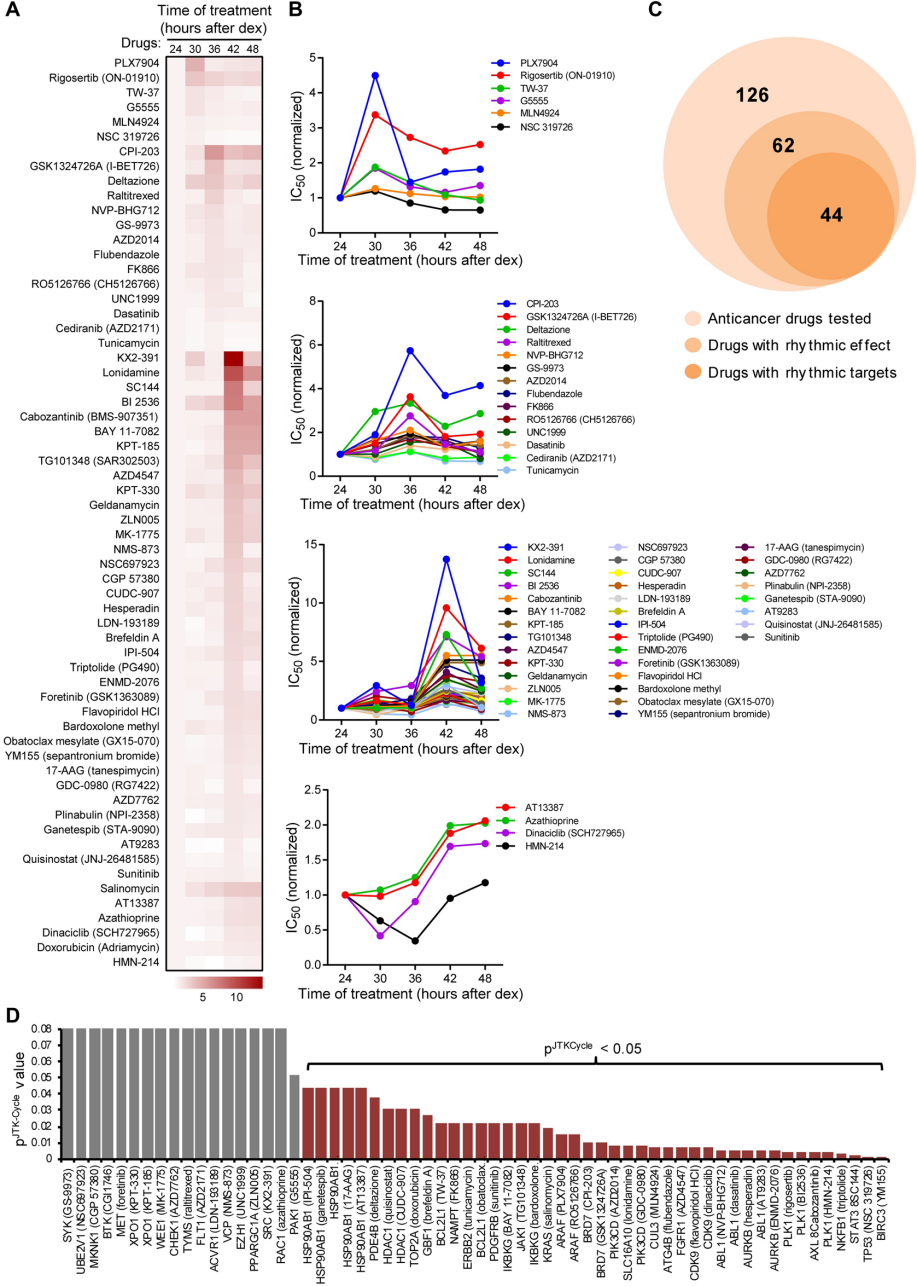
Rhythmic action of anticancer drugs is associated with rhythmic expression of some target genes. (**A**) The heatmap represents anticancer inhibitors (62) exhibiting rhythmic patterns of drug sensitivity from the drug panel (126) tested in U2OS cells. Cells were synchronized by 100 nM dex in staggered fashion, 6 hours apart from each other as shown in fig. S1. Drug treatment was at a single time point 24 hours after the last group was synchronized with dex (thus, at 24, 30, 36, 42, or 48 hours following dex). (**B**) Anticancer drugs show peaks of IC_50_ value at different times. Each line graph represents drugs that are most ineffective at the same time of day (30, 36, 42, and 48 hours) following synchronization of endogenous rhythms with dex. (**C**) Circular plots depict the number of rhythmically acting cancer inhibitors, including those that target rhythmically expressed genes (44; dark orange), of the total tested (126; light orange). (**D**) The bar graph shows rhythmically expressed genes (in brown) targeted by rhythmically acting anticancer inhibitors. Rhythmicity was determined by *P*^JTKCycle^ (*P* < 0.05) and based on ad hoc analysis of RNA sequence data of dex-synchronized U2OS cells across circadian time (see fig. S2A). Gray bars indicate nonrhythmic genes targeted by drugs that act rhythmically (*P*^JTKCycle^ > 0.05).

IC_50_ values at different times of day were normalized to those at the first time point of drug treatment (24 hours after dex). Of the compounds assayed, 62 exhibited rhythmic patterns of efficacy with IC_50_ values peaking at different times (30, 36, 42, and 48 hours relative to dex treatment) (Fig. 1, A and B, and table S1). Based on previous transcriptome profiling that showed circadian oscillations of many putative drug targets (*8*, *9*), we predicted that genes targeted by our rhythmically acting drugs would be expressed with a rhythm. To determine whether this was the case, we examined the cycling transcriptome data of U2OS cells synchronized in a similar fashion as here (*15*). The comparative drug target gene analysis revealed that more than half the genes targeted by our rhythmically acting drugs (44 of 62) exhibit significant periodic expression (*P*^JTKCycle^ < 0.05) (Fig. 1, C and D, and fig. S2A). This may be an underestimate as *wee1*, previously shown to cycle in expression (*22*), was not detected as a cycler in our analysis of U2OS cells (Fig. 1D). Follow-up analysis did not reveal a clear phase correlation between the timing of drug efficacy and target gene expression, although drugs tended to be more effective at the trough of target gene cycling, likely because complete inhibition was more readily achieved at lower levels of target (fig. S2, B and C). However, we acknowledge that target protein accumulation may be delayed relative to transcript levels. Nevertheless, these results suggest that mechanisms beyond the phase of target gene mRNA oscillation contribute to rhythmic cytotoxicity of the antitumor compounds.

### Time-of-day specificity of many drugs is driven by the circadian clock

**Fig. 2:**
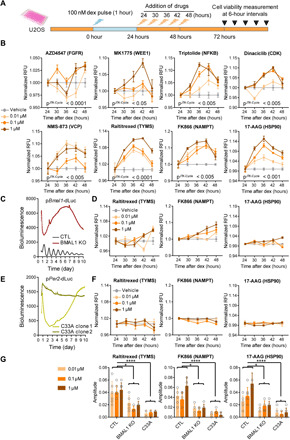
Rhythmic action of anticancer drugs is largely dependent on the circadian clock. (**A**) Schematic of the experimental schedule to validate time dependence of candidates from the drug screen. Twenty-four hours after 1-hour dex synchronization (100 nM, light blue bolt), CRISPR scramble control reporter U2OS cells were treated with vehicle or candidate drugs (0.01 to 1 μM, red bolt) at 6-hour intervals over the course of 24 hours and subjected to an Alamar Blue cell proliferation assay at the indicated time points (48 hours later for each sample). (**B**) Graphs of experimental procedures in (A) to show time-dependent antiproliferative effects of the indicated anticancer drug candidates at various doses (0.01, 0.1, and 1 μM). The candidate drug targets were indicated in the parentheses. *P*^JTKCycle^ < 0.05 denotes results of JTK-Cycle analysis, which detected a significant 24-hour rhythm in the action of AZD4547, MK1775, triptolide, dinaciclib, NMS-873, raltitrexed, FK866, and 17-AAG (details are in data file S3). Data were normalized to represent the average ± SD; *n* = 3 for each time point. RFU, relative fluorescence units. (**C**) Bioluminescence recordings of *Bmal1* promoter (p*Bmal1*-dsLuc) luciferase rhythms in CRISPR scramble control reporter U2OS cells (CTL, dark gray) and BMAL1 CRISPR knockout (BMAL1 KO, brown red) are shown. (**D**) The graphs show compromised rhythms of antiproliferative action of the indicated drugs in BMAL1 null cells. Several doses were tested. (**E**) Bioluminescence recordings of Per2 promoter (p*Per2*-dsLuc) luciferase rhythms in RB mutant C33A cell clones (clone 1: light gray green and clone 2: dark gray green) are shown. (**F**) The graphs show compromised rhythms of antiproliferative action of the indicated drugs in RB mutant C33A cells at various doses. (**G**) JTK-cycle analysis of rhythm amplitude for the time courses shown in (B), (D), and (F). *P < 0.05, ****P < 0.0001.

To validate the temporal regulation of cytotoxicity, we selected a subset of drugs displaying relatively strong rhythms of action with different peak phases in the HTS (Fig. 1 and table S1). We exposed U2OS cells to varied concentrations (0.01, 0.1, and 1 μM) of the selected drugs at different times of day (24, 30, 36, 42, and 48 hours), starting at 24 hours after dex-induced circadian synchrony (Fig. 2A). In the subsequent cell viability assay, we observed that 8 of 10 drugs assayed (AZD4547, MK1775, triptolide, dinaciclib, NMS-873, raltitrexed, FK866, and 17-AAG) showed significant rhythms of cytotoxicity at several doses, but two drugs (PLX7904 and SC144) did not (Fig. 2B and fig. S3A). To investigate whether the rhythmic drug sensitivity relies on the core circadian clock, we knocked out BMAL1, an essential clock gene, using CRISPR-Cas9 in U2OS cells expressing a *Bmal1* promoter–driven luciferase reporter (p*Bmal1-dLuc*) (fig. S4A). Real-time bioluminescence of the clock reporter showed robust circadian rhythms in control cells (CTL), but rhythms were abolished in BMAL1-deficient cells (BMAL1 KO) (Fig. 2C). In parallel, when exposed to the timed drug delivery scheme as above (Fig. 2A), clock gene disruption substantially blunted the cytotoxicity rhythms of the tested drugs (raltitrexed, FK866, 17-AAG, and MK1775) (Fig. 2, D and G, and figs. S3B and S5). Notably, rhythmic cytotoxicity of 17-AAG was recapitulated in forskolin (fsk) or serum shock (SS) synchronized cells, and in all these cases, it required a functional circadian clock (fig. S5). Rhythms of drug sensitivity were similarly dampened in cervical carcinoma cells (C33A) that carry an inactivating mutation in the retinoblastoma (RB) tumor suppressor and lack cellular oscillations of Per2 promoter–driven luciferase (p*Per2-dLuc*) (Fig. 2, E to G). Together, these results strongly suggest that rhythmic cytotoxic actions of some anticancer inhibitors are dependent on the circadian clock.

### Drugs targeting HSP90 have higher cytotoxicity at specific times of day

**Fig. 3:**
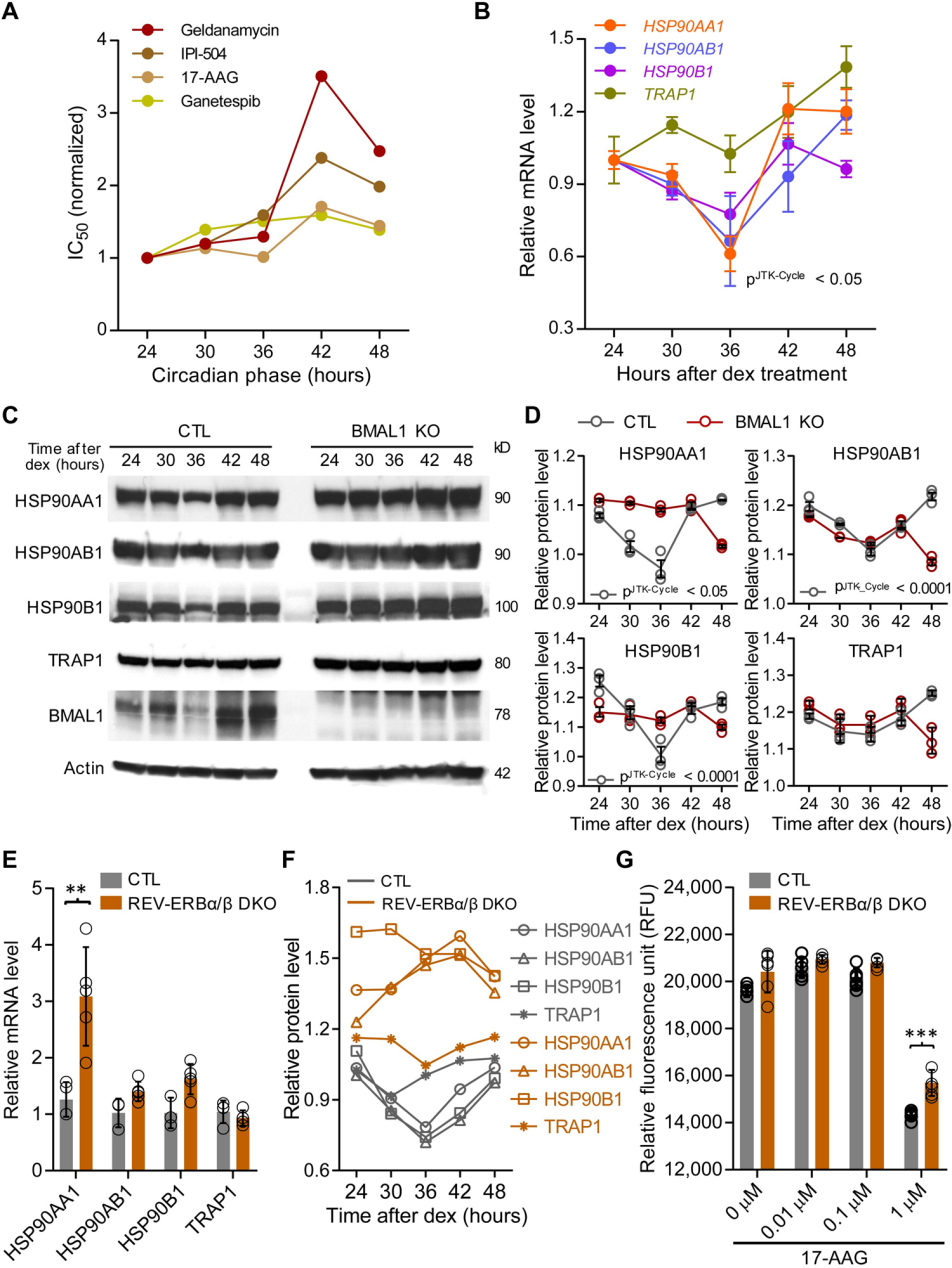
Rhythmic effect of HSP90 inhibitors is associated with circadian expression of HSP90 genes. (**A**) Line graphs are replotted on the basis of the primary screening results in Fig. 1 to show that several HSP90 inhibitors are more effective at inhibiting cell proliferation at a specific time of day. The *x* axis depicts circadian phase as determined by the timing of dex addition (see Materials and Methods). (**B**) Temporal mRNA expression of the indicated HSP90 isoforms of dex-synchronized U2OS cells across circadian time (*15*). *P*^JTKCycle^ < 0.05 indicates significant rhythms of HSP90AA1, HSP90AB1, and HSP90B1 (details are in data file S3). (**C**) Western blot analysis of the indicated HSP90 isoforms and BMAL1 in control (CTL) and BMAL1 knockout U2OS (BMAL1 KO) cells collected every 6 hours for 24 hours after dex stimulation. (**D**) Statistical analysis of the Western blot data in (C) showing protein abundance over time in CTL (gray circle) and BMAL1 KO (red brown circle) cells. JTKCycle identified a significant 24-hour rhythm in the expression of HSP90AA1, HSP90AB1, and HSP90B1 in CTL cells. Data were normalized to actin and are represented as means ± SEM from *n* = 3 independent experiments. (**E**) mRNA expression of the indicated HSP90 isoforms in control (CTL) and REV-ERB α/β double-knockout U2OS cells (REV-ERBα/β DKO). **P < 0.005; two-way ANOVA with Bonferroni’s multiple comparisons test. Data are shown with the means ± SD; *n* = 3 to 6 in each genes. (**F**) Line graphs, depicting means based on the data in fig. S6B, show expression profiles of the indicated HSP90 isoforms in CTL (gray) and REV-ERBα/β DKO (light brown) cells. (**G**) Bar graphs depict cell viability assays performed 48 hours after 17-AAG treatment in CTL (gray bar) and REV-ERBα/β DKO (light brown bar) cells. ***P < 0.005. Data show means ± SD; *n* = 6 for all doses.

We found that a subset of antitumor inhibitors (geldanamycin, IPI-504, 17-AAG, and ganetespib) targeting the same molecule, namely HSP90, exhibited similar rhythms of cytotoxicity (Fig. 3A). The Hsp90 chaperone complex stabilizes several oncoproteins, so drugs targeted to HSP90 can inhibit multiple targets to induce tumor cell cytotoxicity and apoptosis (*23*). In mammals, four Hsp90 paralogs (Hsp90aa1, Hsp90ab1, Hsp90b1, and Trap1) with varied molecular structures and subcellular localization play an integral function in regulating protein quality (*24*). Our temporal transcriptome analysis of dex-synchronized U2OS cells (*15*) revealed significant oscillations of *HSP90AA1*, *HSP90AB1*, and *HSP90B1* but not *TRAP1* (Fig. 3B). Time-course Western blot analysis recapitulated a similar pattern of temporal oscillations in the protein abundance of HSP90AA1, HSP90AB1, and HSP90B1 (Fig. 3, C and D, and fig. S6). However, rhythmic expression of the HSP90 proteins was completely lost in BMAL1-deficient cells (BMAL1 KO) compared with the control (CTL) (Fig. 3, C and D, and fig. S6). Consistent with our results in U2OS cells, post hoc analysis of published circadian gene expression data (*25*, *26*) showed marked oscillations of *Hsp90aa1*, *Hsp90ab1*, and *Hsp90b* but not *Trap1* in various mouse tissues including liver (fig. S7). Rhythmic Hsp90 transcripts (*Hsp90aa1*, *Hsp90ab1*, and *Hsp90b*) oscillate in phase with *Bmal1*, not *Per2*, in liver tissue (fig. S7, B to D). This suggests rhythmic control of HSP90 genes by the transcriptional regulatory mechanism that drives Bmal1 cycling, namely the nuclear receptors ROR/REV-ERB, which act on the same response element (RORE). In support of this idea, cistromic analysis of previous genome-wide chromatin immunoprecipitation sequencing (ChIP-seq) data (*27*) indicates relatively strong binding of Rev-erbα to the promoter regions of *Hsp90aa1*, *Hsp90ab1*, and *Hsp90b* in mouse liver (fig. S8).

To test for a direct role of REV-ERB transcriptional repressors in the regulation of HSP90 expression, we generated U2OS cells deficient in both REV-ERB α and REV-ERB β (REV-ERBα/β DKO) using CRISPR-Cas9 (fig. S4B). U2OS cells lacking the two REV-ERB proteins (REV-ERBα/β DKO) showed higher levels of the HSP90 mRNAs, particularly *HSP90AA1*, and also higher levels of the corresponding proteins (Fig. 3, E and F, and fig. S6). We also examined the pathway.

**Fig. 4:**
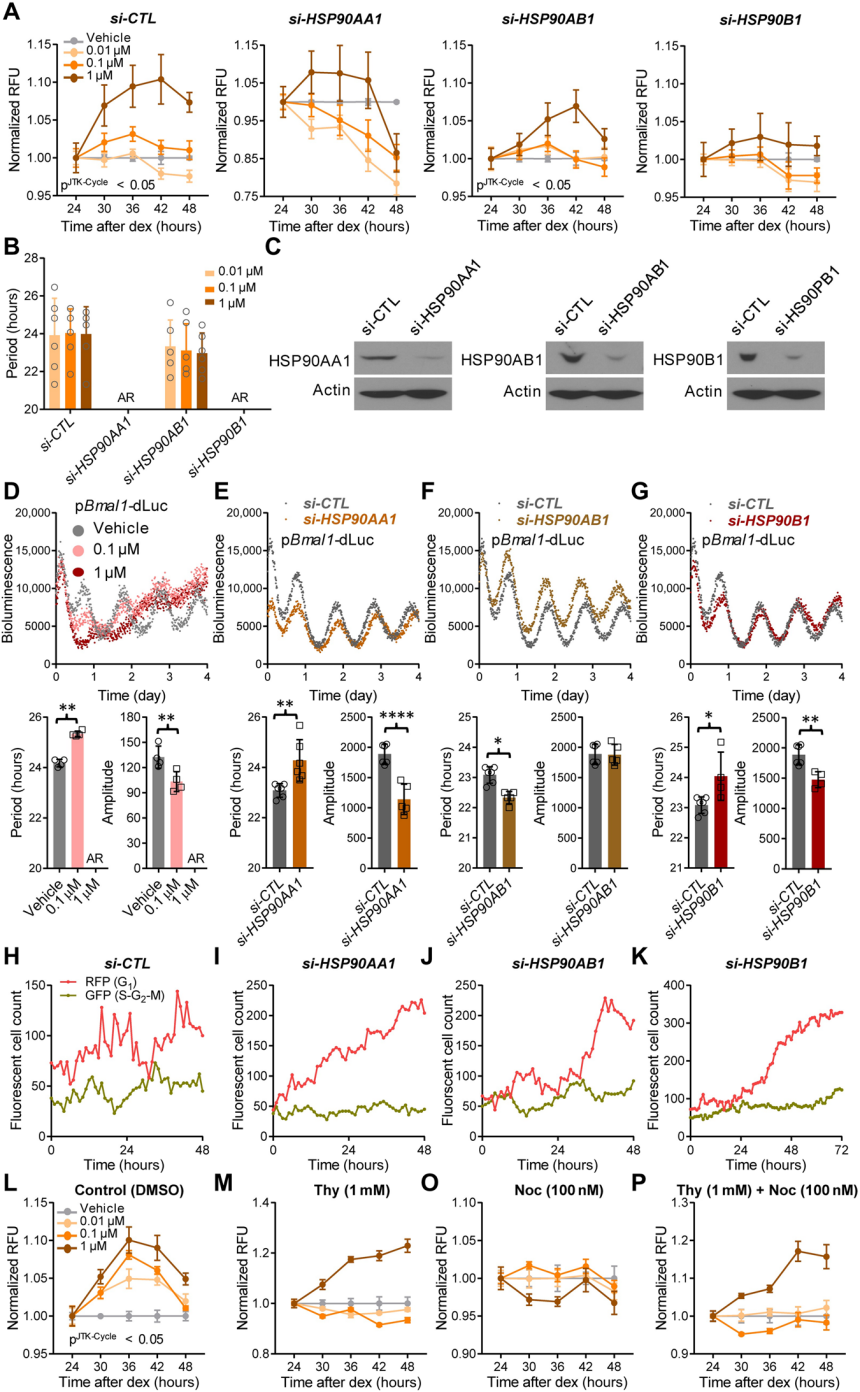
Isoforms of HSP90 that mediate temporal drug sensitivity affect circadian rhythms and the cell cycle. (**A**) Graphs show time-of-day–dependent cytotoxic effect of 17-AAG delivered to dex-synchronized U2OS cells treated with control (*si-CTL*) or siRNAs targeting the indicated HSP90 isoforms. JTKCycle detected a significant 24-hour rhythm in 1 μM drug-treated si-CTL and si-HSP90AB1 samples (details are in data file S3). (**B**) Period analysis of temporal 17-AAG cytotoxicity data shown in (A) using the Biodare2 rhythm analysis program (https://biodare2.ed.ac.uk/). AR, arrhythmic. (**C**) Western blot analysis to verify efficiency of siRNAs targeting the indicated HSP90 isoforms. (**D** to **G**) Bioluminescence recordings of a *Bmal1* promoter–luciferase reporter (p*Bmal1*-dsLuc) in U2OS cells treated with vehicle (gray) or 17-AAG at the indicated doses (D) or 48 hours after transfection of control siRNA (*si-CTL*, gray) or siRNAs targeting the indicated HSP90 isoforms (E to G) (top). Graphs below present period (left) and amplitude (right) analysis results of the top data panels. *P < 0.05, **P < 0.005, and ****P < 0.00001 by Biodare2 analysis. Data show means ± SD; *n* = 3 for all samples. (**H** to **K**) Real-time cell analysis of U2OS cells stably expressing FUCCI cell cycle indicators [RFP (G_1_), dark pink; GFP (S-G_2_-M), gray green) 48 hours after transfection of control siRNA (*si-CTL*) (H) or siRNAs targeting the indicated HSP90 isoforms (I to K) (top). (**L** to **P**) DMSO (control) or cell cycle arrest inducers such as thymidine [Thy (1 mM), G_1_-S boundary arrest] and nocodazole [Noc (100 nM), G_2_-M arrest] were delivered alone or in combination as indicated to block cell cycles in U2OS cells before timed 17-AAG treatment and cell viability analysis as in Fig. 2A. Data are normalized to represent average ± SD; *n* = 6 per time point. *P*^JTKCycle^ detected a significant 24-hour rhythm in control cells treated with 0.01 μM, 0.1 mM, or 1 μM 17-AAG (details are in data file S3).

To determine how the different HSP90 isoforms contribute to the temporal cytotoxic effect of their inhibitor, we silenced each of the gene paralogs in U2OS cells using small interfering RNAs (siRNAs) and treated the cells with 17-AAG at 6-hour intervals (24, 30, 36, 42, and 48 hours) following dex synchronization (Fig. 4A). In parallel, the knockdown efficiencies of the individual siRNAs were confirmed by Western blot analysis (Fig. 4C). Cytotoxic rhythms were substantially dampened by knockdown of *HSP90AA1 or HSP90B1* but not *HSP90AB1* (Fig. 4, A and B). Time-of-day variation of drug sensitivity was still robust in the expression of HSF1, a previously known heat shock–responsive transcriptional inducer of HSP90 genes (*24*), and we found that it cycles antiphase to the HSP90 paralogs and BMAL1 in control cells (fig. S6). HSF1 levels were increased across time in BMAL KO cells, while in REV-ERB DKO cells, cycling was abrogated without a notable difference in levels. Given that both knockouts affected HSP90, these findings suggest that HSF1 is not directly involved in the clock gene–mediated regulation of HSP90 under non–thermal stress conditions. Consistent with increased levels of the prosurvival HSP90 molecules, REV-ERBα/β DKO cells exhibited relatively strong drug resistance compared with control cells (CTL) when treated with 17-AAG, a potent tumor-specific HSP90 inhibitor (Fig. 3G). Moreover, the rhythmic toxicity of 17-AAG observed in CTL cells was impaired in REV-ERBα/β DKO cells as it was in BMAL1 KO cells, regardless of which method was used to synchronize cellular rhythms (fig. S5). These results together suggest that a subset of HSP90 genes is directly controlled by the circadian molecular machinery and thereby likely confers a cyclic response to drugs targeting control siRNA–treated cells (*si-CTL*). To address the mechanism of HSP90-mediated drug response rhythms, we investigated the effect of HSP90 siRNAs on circadian rhythms using the p*Bmal1-dLuc* reporter. Real-time bioluminescence recording showed that RNA interference (RNAi)–mediated depletion of *HSP90AA1* or *HSP90B1* significantly prolongs circadian period and dampens the amplitude of rhythms, an effect also observed in cells treated with the HSP90 inhibitor 17-AAG (Fig. 4, D, E, and G). However, *HSP90AB1*-depleted cells exhibited comparable rhythms, albeit with a shorter period, to control siRNA–treated cells (Fig. 4F). Concomitant with these results, knockdown of HSP90 isoforms exerted different effects on the expression of core clock proteins (PER2, CRY1, CRY2, CLOCK, and BMAL1) (fig. S9A). Notably, the abundance of CRY1, but not CRY2, was decreased upon knockdown of HSP90AA1 relative to HSP90AB1, despite the marked increase of BMAL1 when either of the genes was silenced (fig. S9A). Immunoprecipitation analysis revealed that HSP90AA1, but not HSP90AB1, interacts with CRY1, but not CRY2, suggesting that CRY1 is a direct substrate of HSP90AA1 for protein stability (fig. S9B). These results implicate specific rhythmically expressed isoforms of HSP90 in cyclic drug toxicity and also suggest feedback of these isoforms on clock proteins.

### Effects of HSP90 on rhythmic drug sensitivity are mediated through changes in the cell cycle

The circadian clock interacts with the cell cycle on multiple levels, so we sought to determine whether this interaction is relevant for temporal cellular responses to antitumor agents (*28*). We assayed the cell cycle though fluorescence ubiquitination-based cell cycle indicator (FUCCI) imaging, which uses fluorescent probes to demarcate stages of the cell cycle [hCDT1-mKO2 marks cell growth (G_0_-G_1_) with red fluorescent protein (RFP), and hGeminin-mAG labels DNA replication (S) and cell division (G_2_-M) phases with green fluorescent protein (GFP)] (*29*). We stably introduced the probes into U2OS cells and monitored the cell cycle following RNAi knockdown of HSP90 molecules (fig. S10A). Real-time analysis of cells revealed that the cell cycle was relatively well synchronized in control siRNA–treated cells (*si-CTL*), such that the FUCCI sensors representing G_0_-G_1_ and S-G_2_-M, respectively, were antiphasic to each other (Fig. 4H). However, this rhythmic pattern of the cell cycle was largely abrogated in response to knockdown of *HSP90AA1 or HSP90B1* but not *HSP90AB1* (Fig. 4, I to K). These observations prompted us to test the direct impact of the cell cycle on the rhythm of drug action. To this end, we treated cells with thymidine and nocodazole, which arrest the cell cycle at the G_1_-S and G_2_-M transitions, respectively, before the timed administration of 17-AAG. Dimethyl sulfoxide (DMSO)–treated control cells (DMSO) exhibited dose- and time-dependent effects of the HSP90 inhibitor at different concentrations (0.01, 0.1, and 1 μM) (Fig. 4L and fig. S11A). However, pretreatment with thymidine (Thy; 1 mM) and/or nocodazole (Noc; 100 nM) (Fig. 4, M to O) abrogated the rhythm of the drug response, although it did not affect dose-dependent cytotoxicity at the different time points (fig. S11, B to D). Despite their effect on the timing of the drug response, these cell cycle blockers also did not affect circadian rhythmicity in cells (fig. S11, E to H). On the other hand, a functional circadian clock was indispensable for normal cell cycle regulation because the cell cycle synchrony evident in control FUCCI reporter cells (CTL) was completely lost in BMAL1-deficient cells (BMAL1 KO) (fig. S10B). These data suggest that, in addition to an intact circadian clock, temporal regulation of HSP90 inhibition requires HSP90-dependent progression of the cell cycle.

### HSP90 inhibition has time-of-day–specific effects on a mouse melanoma

**Fig. 5:**
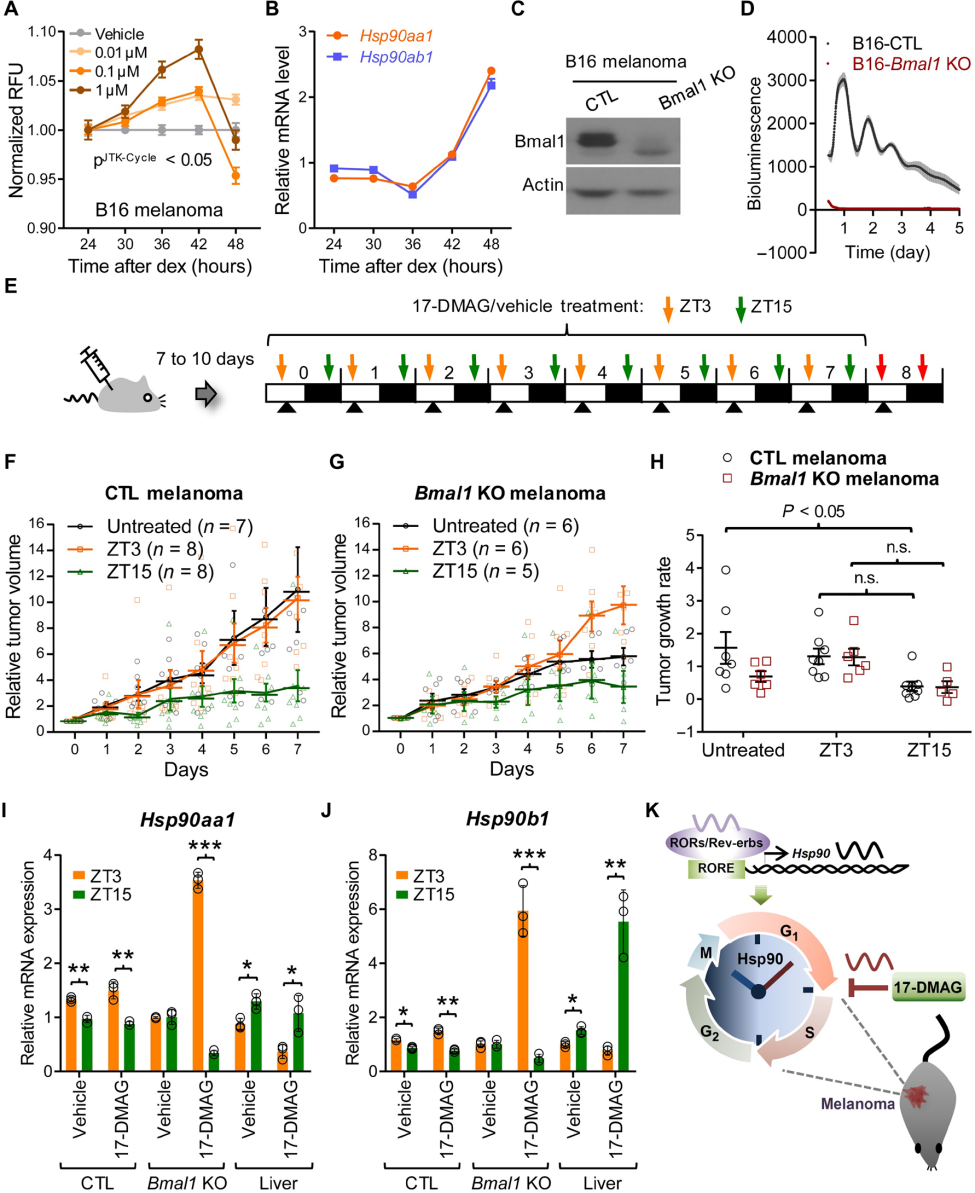
17-DMAG exerts time-of-day–specific anticancer activity on a mouse melanoma. (**A**) Time-of-day–dependent cytotoxicity of different doses of 17-AAG in dex-synchronized B16 melanoma cells. *P*^JTKCycle^ < 0.05 indicates significant cytotoxic rhythms at 0.1 and 1 μM 17-AAG. (**B**) mRNA expression of Hsp90aa1 (red) and Hsp90ab1 (blue) in dex-synchronized B16 melanoma cells. (**C**) Western blot analysis of CRISPR scramble (CTL) and *Bmal1* knockout (*Bmal1* KO) B16 cells using indicated antibodies. (**D**) Bioluminescence recordings of dex-synchronized control CTL (black) and *Bmal1* KO (red) cells stably expressing *Per2* promoter–driven luciferase reporter (p*Per2*-dLuc). (**E**) Seven or 10 days after subcutaneous injection of CTL or *Bmal1* KO cells (1 × 10^6^), mice were orally administered 17-DMAG at ZT3 (orange arrow) or ZT15 (green arrow). Black arrowheads denote times of tumor measurement. Red arrows indicate sampling of tumor and liver tissues from mice sacrificed at ZT3 or ZT15 for gene expression analysis. (**F** and **G**) Time-dependent effects of 17-DMAG administration on CTL (F) or *Bmal1* KO (G) tumor growth in mice; untreated (black circle), treated at ZT3 (orange rectangle), and treated at ZT15 (green triangle). (**H**) Quantification of relative tumor growth rate calculated from linear regression by fitting a linear equation to the normalized data panels in (E) and (F). *P < 0.05, two-way ANOVA and Tukey’s multiple comparisons test. Data are means ± SEM. (**I** and **J**) *Hsp90aa1*(I) and *Hsp90b* (J) expression in CTL or *Bmal1* KO melanoma and liver tissues from mice as described in (D). *P < 0.05, **P < 0.005, and ***P < 0.0001, one-way ANOVA and Tukey’s multiple comparisons test. Data are means ± SD; *n* = 3. (**K**) Schematic depicts circadian regulation of Hsp90 by RORs/Rev-erbs, via the specific response elements (RORE), and its action in the cell cycle to confer temporal specificity to the antimelanoma effects of 17-DMAG. n.s., not significant.

HSP90-targeting drugs have been tested and considered in the treatment of several types of malignant melanomas, either on their own or as a complementary therapeutic strategy, in preclinical studies and clinical trials (*30*). To determine whether hsp90 inhibitors act on melanoma in a time-dependent fashion, we used the B16 mouse skin cancer cell line, which is highly metastatic but has intrinsic circadian function (*17*). Following dex synchronization, the melanoma cells displayed a circadian pattern of drug sensitivity (*P*^JTKCycle^ < 0.05) in response to an HSP90 inhibitor and also showed rhythmic expression of a subset of Hsp90 genes (*Hsp90aa1* and *Hsp90ab1*) (Fig. 5, A and B). To determine whether time dependence is maintained in the intact animal, we introduced the B16 melanoma cells into mice via subcutaneous injection (fig. S12A). After detectable tumors developed, we gave daily oral treatments of 17-DMAG (10 mg/kg), a water-soluble analog of 17-AAG, to the mice for 7 days at 3 hours after lights on [zeitgeber time 3 (ZT3)] (morning) or 3 hours after lights off (ZT15) (night) (fig. S12A). We observed a significant reduction of tumor growth rate in mice receiving the drug at ZT15, but not those treated at ZT3, despite minimal change in body weight between the experimental and control groups of mice during the timed drug treatment schedule (fig. S12, E to G). Notably, locomotor activity rhythms were not significantly altered by 17-DMAG treatment (fig. S12, A to D). Western blot analysis showed relatively higher protein expression of Hsp90aa1 and Ccnb1, which marks proliferating cells, in tumor tissues collected at ZT3 relative to ZT15 (fig. S12H), suggesting that the time-restricted antitumor effect of the hsp90 inhibitor is likely to be modulated by the daily expression of HSP90 in mouse melanoma tissues.

To determine whether time-modulated drug activity on the mouse melanoma was core clock dependent, we generated CRISPR-control (B16-CTL) and *Bmal1* knockout (B16-*Bmal1*-KO) melanoma cells stably expressing *Per2* promoter–driven luciferase (p*Per2-dLuc*) (Fig. 5C). Control melanoma cells (B16-CTL) exhibited marked circadian oscillation of the clock reporter activity upon dex synchronization, but rhythms were completely abrogated in *Bmal1*-deficient cells (B16-*Bmal1*-KO) (Fig. 5D). Next, we injected the control (B16-CTL) and *Bmal1* KO melanoma cells (B16-*Bmal1*-KO) into mice subcutaneously and subjected them to drug treatment after detectable tumor formation as described above (Fig. 5E). As observed in the prior data (fig. S12, E and F), daily treatment with 17-DMAG at ZT15, but not at ZT3, significantly suppressed growth of the control tumor (Fig. 5, F and H). In addition, both quantitative polymerase chain reaction (qPCR) and Western blot analysis revealed relatively higher expression of Hsp90 genes, particularly Hsp90aa1 and Hsp90b1, in the control tumor tissue (CTL) collected at ZT3 versus ZT15; however, this daily variation was not observed in *Bmal1* KO melanoma tissue (Fig. 5, I and J, and fig. S13D). As with the U2OS cell data, time-of-day variation of HSF1 was observed in control mouse tumor tissues with higher levels at ZT15 than at ZT3, again antiphasic to Hsp90aa1 and Hsp90b1 (fig. S13D). Notably, daily fluctuations of HSF1 are not derived solely from circadian clock control because similar variations occur in Bmal1 KO tumors (fig. S13D).

In contrast to the control tumor, the *Bmal1* knockout melanoma (B16-*Bmal1*-KO) was not significantly suppressed by the HSP90 inhibitor at ZT15 (Fig. 5, G and H). It is possible that the slower growth rate of this tumor contributed to blunting the effect of the drug. Unexpectedly though, Bmal1-deficient tumors responded to drug administration at ZT3 with a notably higher growth rate, perhaps because of a substantial induction of Hsp90 in the tumor (Fig. 5, G, I, and J); although not detected in steady-state protein expression (fig. S13D), increased HSP90 likely accounts for increased growth and the resulting apparent (albeit not significant) difference between ZT3 and ZT15 in *Bmal1* knockout tumors.

Pharmacokinetic analysis of the drug in wild-type mice showed overall similar parameters between ZT3 and ZT15 except for slightly earlier drug absorption at ZT15 (fig. S13C and table S2). This may explain better efficacy of the drug in terms of cleaved caspase 3 fragments, a 17-DMAG–dependent apoptosis marker (*31*), at ZT15 in both control and *Bmal1* KO groups (note that even the *Bmal1* KO is in a wild-type host). Overall, our animal data support the cell culture findings, indicating that an anti-HSP90 inhibitor is effective at a specific time of day on a wild-type tumor.

## DISCUSSION

Circadian rhythms are thought to modulate cellular responses to xenobiotic cues over a 24-hour day (*32*). Using cell-based pharmacological approaches to assess the influence of time of day (chronopharmacology), we demonstrate that a preserved circadian clock in cancer cells has a critical role in mediating responses to anticancer agents. We further demonstrate that drugs targeting HSP90 act in a time-dependent manner due to the modulation of the cell cycle by specific rhythmically expressed HSP90 isoforms (Fig. 5K).

It is commonly thought that circadian functions in cancer cells are compromised or deregulated, at least in part because of high expression of oncogenic Myc or Ras (*12*, *13*). However, some cancer cells, such as U2OS human bone tumor and B16 mouse melanoma models, can be induced with dex to display circadian rhythms despite their highly tumorigenic and metastatic potential (*15*, *17*, *33*). We previously showed that U2OS cells, derived from an osteosarcoma, also exhibit temporal responses to antitumor agents (*15*). In line with these experimental tumor models, patient-derived cancer stem cells (CSCs) of glioblastoma or acute myeloid leukemia display robust circadian rhythms despite high MYC expression levels (*34*, *35*). Notably, recent pharmacological studies using glioblastoma stem cell models have focused on the tumor-specific cytotoxic effect of small molecules directly targeting core circadian regulators (*34*, *36*). There is growing evidence for the presence of functional clocks in CSCs, which are generally considered the key drivers of tumor progression and drug resistance. However, while circadian regulators in these CSCs promote proliferation, clock proteins act as tumor suppressors in the context of many tumors (*37*–*39*).

Although previously implicated in circadian rhythms (*40*), HSP90 is typically thought to function in a housekeeping capacity. Our findings suggest that it has an important function in linking the clock to the cell cycle and mediating time-of-day–specific drug responses. Antitumor effects of HSP90-targeted drugs can be mediated by several isoforms, prompting us to identify the isoform relevant for time-of-day specificity. Isoforms of Hsp90 differ in terms of gene expression and subcellular localization as well as their roles in cellular differentiation and development (*23*). Although mammalian Hsp90AA1 and Hsp90AB1 have similar structure and cytosolic expression, Hsp90AA1 is induced by external cues to enhance oncogenic signaling responses and cell cycle progression, while Hsp90AB1 is expressed constitutively, mostly for cellular maintenance (*24*). Similar to the pro-oncogenic role of Hsp90AA1, Hsp90B1, an endoplasmic reticulum–localized subtype of Hsp90, interacts with multiple mitogenic and prosurvival factors to promote cancer development and metastasis (*41*). Consistent with the distinctive cellular functions of the HSP90 isoforms, our present data show that, despite the cyclic expression of three different HSP90 isoforms, selective silencing of either HSP90AA1 or HSP90B1, but not HSP90AB1, substantially disrupts circadian-regulated cell proliferation and drug cytotoxicity (Figs. 3 and 4). These results also support our in vivo observation that mouse melanoma tissues exhibit time-of-day–dependent responses to an HSP90 inhibitor in phase with the diurnal cycling of Hsp90aa1 and Hsp90b1 (Fig. 5, F to J, and figs. S12, E to H, and S13D). Pharmacodynamic studies show that the best-studied HSP90 inhibitors (e.g., 17-AAG and 17-DMAG) have higher affinity for HSP90AA1 than HSP90B1 (*42*). Considering all these factors, we propose that HSP90AA1 is the isoform responsible for mediating temporal action of drugs targeted to the HSP90 pathway.

HSP90 genes are regulated in response to heat shock or proteotoxic stress by the transcription factor HSF1. Our in vitro and in vivo expression analysis shows that HSF1 exhibits daily fluctuations that are antiphasic to protein oscillations of HSP90 paralogs and are also differently affected in clock gene knockout cells or tumor tissues (figs. S6 and S13D). Thus, we reason that HSF1 is not directly involved in circadian-regulated HSP90 expression in our tumor models, particularly under nonstress conditions. An earlier study showed that HSF1 deficiency abolishes heat shock but not dex-synchronized circadian m*Per2* rhythms in mouse embryonic fibroblasts (*43*). Given this, it is possible that HSF1 mediates sensitivity to HSP90 inhibitors in cells synchronized with a heat shock protocol, although we did not test this idea.

Temporal effects of the drug are lost in cells lacking the BMAL1 clock protein, although cytotoxicity of the drug persists in these cells (Fig. 2). In addition, C33A cervical carcinoma cells do not support cycling of a circadian reporter and lack temporally modulated responses to an anti-HSP90 drug (Fig. 2, E and F). Furthermore, genetic deletion of Rev-erbα/β, a circadian repressor that potentially contributes to Hsp90 cycling based on our analysis of global gene cycling and ChIP-seq data, also eliminates rhythms of drug action and increases resistance to an HSP90 inhibitor; this is likely due to enhanced cell proliferation and survival as a result of up-regulated HSP90 (Fig. 3 and figs. S5 to S8). Effects on HSP90 may explain, in part, why pharmacological activation of REV-ERBs reduces oncogene-driven cancer cell proliferation and growth (*36*, *44*).

At the same time, our findings implicate synchrony of the cell cycle as an important mediator of temporal drug action. Notably, the C33A cervical carcinoma cells mentioned above also affect the cell cycle as they express a truncated unstable RB protein, a negative regulator of the G_1_ to S cell cycle transition. Experimental and computational modeling studies support the idea of reciprocal interactions between the circadian clock and the cell cycle (*45*–*47*). We suggest that HSP90 is an important mediator of such interactions. Several proteomic studies and our data here show that Hsp90 molecules functionally interact with core circadian clock components (e.g., Rev-erbα, Gsk3b, Bmal1, and Cry1) and cell cycle regulators (e.g., Cdk4/6, Ccnd1, and Wee1), most of which, in turn, are clock controlled (fig. S9B) (*40*, *48*, *49*). Not unexpectedly, genetic perturbation of HSP90 leads to alterations of both circadian rhythms and cell cycle synchrony accompanied by loss of rhythmic cellular sensitivity to an HSP90 inhibitor (Fig. 4). Nevertheless, given that knockdown of specific isoforms of HSP90 completely eliminates the rhythm of drug sensitivity but only mildly affects periodicity of the clock, it is likely that HSP90 acts downstream of the clock to regulate the cell cycle and thereby the cycling of drug sensitivity. Supporting this idea, cell cycle arrest reagents abrogated time-of-day cytotoxicity of the HSP90 inhibitor without significantly affecting circadian rhythms (fig. S11). On the other hand, disruption of the circadian clock severely impaired cell cycle synchrony and rhythmic drug effects (Fig. 2 and figs. S3B, S5, and S10B). We propose that rhythmic drug action arises in large part from cell cycle synchrony driven by circadian-regulated genes. Thus, while cycling of the target is necessary, higher efficacy of the drug at a specific time of day does not just result from altered (e.g., lower) levels of the target at that time of day but rather the effect of the cycling target on the cell cycle. Regulated progression of the cell cycle was also implicated in the temporal action of the mTOR inhibitor everolimus, but in that case, the regulation was suggested to be independent of the circadian clock (*50*). In support of the idea that the clock within the tumor is important, a *Bmal1* knockout tumor in a wild-type host is not protected by the HSP90 inhibitor, although it responds to the inhibitor in a different way (see below).

Consistent with circadian effects on the cell cycle, inactivation of Bmal1 was recently shown to act through cyclin B1 to delay the G_2_-M transition and thereby prolong the cell cycle (*51*). In addition, although not demonstrated to mediate effects of the clock on the cell cycle, other cell cycle–regulating factors (e.g., Wee1, Myc, and p21) are cyclically expressed in a BMAL1-dependent manner (*22*, *52*, *53*). Last, we note that several rhythmically acting drugs we identified target cell cycle molecules, particularly those that act on the G_2_-M phase such as cyclin-dependent kinase (CDK) (dinaciclib and flavopridol), Polo-like kinase (BI2536, HMN-214, and rigosertib), Aurora kinase (AURK) (AT9283, ENMD-2076, and hesperidin), Wee1 (MK-1775), and ChK (AZD7762) (Fig. 1 and table S1). We suggest that these and other drugs targeted to the cell cycle will have a time-dependent action on all tumors that contain circadian clocks because of the synchronized cell cycle in such tumors.

Although the efficacy of an anti-HSP90 drug is lost in a Bmal1-deficient tumor, we cannot exclude the possibility that host factors also affect temporal efficacy. Notably, the Bma1-deficient tumor treated with drug at ZT3 showed even more growth than the untreated tumor, likely due to the induction, and thereby higher levels, of HSP90. Given that this effect was not observed in cultured tumor cells, it may reflect circadian regulation elsewhere in the host animal, or it may be driven by the light:dark cycles used to house the animals. Regardless, it underscores the possible contribution of tumor nonautonomous regulation to the temporal action of a drug. In this regard, circadian regulation of xenobiotic metabolism in the host (*32*), in particular through Bmal1 (*54*, *55*), could be relevant for the processing of anti-HSP90 compounds at different times of day (Fig. 5). Our pharmacokinetic and pharmacodynamic studies show small differences between ZT3 and ZT15 in the kinetics of drug absorption and molecular effects of drug (fig. S13, C and D, and table S2), although these are unlikely to explain the effects we report here. Consideration of the different cyclic cues experienced by the organism, ranging from internal circadian signals to external environmental cycles, may facilitate the use of properly timed nontoxic doses of HSP90 inhibitors; however, we acknowledge that defining the optimal dose could be difficult.

Despite extensive efforts to develop highly efficient chemotherapeutic molecules, the vast majority of candidate drugs, including several classes of HSP90 inhibitors, have, so far, failed approval by the U.S. Food and Drug Administration (*56*). Based on our earlier and present studies, we reason that variability in results could have resulted from drug administration at inconsistent phases of either the tumor or host circadian rhythms (*15*). In recent years, several approaches have been implemented to enhance the therapeutic efficacy of Hsp90 inhibitors and other candidate antitumor agents. For instance, HSP90 inhibitors improve chemotherapeutic outcomes in several types of cancers when delivered in combination with standard-of-care platinum antitumor compounds (e.g., cisplatin and oxaliplatin) or epidermal growth factor receptor/vascular endothelial growth factor–targeting immunologic agents (cetuximab, panitumumab, and bevacizumab) (*30*, *57*). Moreover, a number of HSP90 inhibitors, including 17-DMAG, improve immunotherapy of melanoma by enhancing T cell–mediated killing of cancer cells (*58*, *59*). On the other hand, exploiting nanoparticle-based carriers that deliver the drug directly to the tumor has been considered to facilitate bioavailability of HSP90 inhibitors in cancer tissues (*60*). Given our findings, we suggest that nanoparticle delivery systems could be optimized to ensure that the drugs are available at the appropriate time of day with respect to intratumoral rhythms.

## MATERIALS AND METHODS

### Study design

The goal of the study was to investigate time-of-day–specific effects of existing antitumor agents and the mechanisms underlying any such effects. To achieve this in an unbiased manner, a cell-based HTS protocol was designed to determine responses of cancer cells to antitumor agents at different times of day. The rhythmic actions of some drugs were further validated by time-scheduled drug delivery in cells synchronized for circadian rhythms. To elucidate the molecular and cellular bases of rhythmic drug actions, several CRISPR-based knockout or siRNA-mediated knockdown strategies as well as multiple transgenic reporter systems were introduced in cancer cells. The clinical relevance of the in vitro findings was investigated in vivo using a mouse cancer model. For in vivo experiments, each group contained between five and eight mice; this provided enough power and validity to detect biologically relevant phenomena. The studies were not performed under double-blind conditions. All live animal experiments were performed according to protocols (806387) approved by the Institutional Animal Care and Use Committee of the University of Pennsylvania in accordance with guidelines set by the National Institutes of Health. Detailed study design, sample sizes, replicates, and statistical analysis for the above in vitro and in vivo studies are provided in Materials and Methods or the figure legends.

### Drugs and compound management

Drugs were purchased from Selleckchem (Houston, TX, USA) as 10 mM stock solutions in DMSO. Compound plates were made on 384-well plates. Each compound plate contained 44 drugs, which were arrayed in eight-point serial dilutions. Dilutions were prepared in 100% DMSO using the JANUS Automated Workstation (PerkinElmer) and Serial Dilution Tool. Wells in column 1 of each plate were designated as the solvent controls and contained 100% DMSO. Wells in column 2 of each plate were designated as the positive controls and contained 5 mM doxorubicin. Each plate contained 5 μl of compound per well, was stored at −40°C, and thawed a maximum of 10 times.

### High-throughput chrono-pharmacological screening

A total of 1000 cells were plated in a volume of 25 μl per well of the 384-well microplates (Corning 3707). They were plated using a Multidrop Combi Reagent Dispenser (Thermo Fisher Scientific). The microplates were incubated in a humidified chamber overnight at 37°C, 5% CO_2_. This allowed the cells to attach to the bottom of the wells and achieve sufficient confluency (~70%) for dex stimulation.

As the screening was done in an HTS facility that did not permit delivery of so many drugs at multiple times per day, we instead synchronized the cells with dex at different times so they would be staggered in their circadian phases at the single time of drug delivery. Dex treatment of different groups of cells occurred at 6-hour intervals across a 24-hour time period to achieve five time points. At each time interval, 10 μl of dex solution was added to each well of the assay plates using the Multidrop Combi Reagent Dispenser (Thermo Fisher Scientific). This was done using low speed to minimize dislodging of the cells from the wells. The final dex concentration in each well was 100 mM. After the final time point addition of dex, all microplates were further incubated for 24 hours in a humidified chamber at 37°C, 5% CO_2_.

At the commencement of drug treatment, cells in the microplates had been dex-stimulated 24, 30, 36, 42, and 48 hours before drug exposure. Fifty nanoliters of drugs was transferred to the assay plates using a 384-well plate, 50-nl slotted pin tool (V&P Scientific), and the JANUS Automated Workstation. The final eight concentrations of drugs on the assay plates were 10 μM, 3.33 μM, 1.11 μM, 370 nM, 123 nM, 41 nM, 14 nM, and 4.6 nM. The final concentrations for the solvent and positive control compounds were 10 μM. Following drug addition, plates were incubated for 72 hours at 37°C, 5% CO_2_.

The ATPLite Luminescence Assay (PerkinElmer) was used to measure cell viability. Plates were removed from the humidified chamber 1 hour before the assay to equilibrate to room temperature. Twenty microliters of ATPLite was added to each well and incubated at room temperature for 10 min. Luminescence was measured using an ultrasensitive luminescence measurement technology on an EnVision Xcite Multilabel plate reader (PerkinElmer).

### Data analysis for high-throughput chrono-pharmacological screening

Each assay plate was analyzed independently. Z′-factors were calculated on the basis of raw values of DMSO- and doxorubicin-treated wells to determine assay performance and data quality. Z′-factor above 0.5 represented acceptable data. Raw data values of test wells were normalized to DMSO and doxorubicin control wells and expressed as normalized percent inhibition (NPI) using the following formula: NPI = ((DMSO_Ave_ − test well) / (DMSO_Ave_ − doxorubicin_Ave_) × 100). IC_50_ values for each drug were determined using nonlinear fit with variable slopes (GraphPad Prism 7) of NPI and log_10_ concentration values. Heatmaps were generated using GraphPad Prism 7.

The IC_50_ values of compounds that extrapolated outside the concentration range we tested were manually assigned IC_50_ values. For inactive or weakly inhibitory compounds (i.e., computed IC_50_ ≥ 25 μM), we assigned IC_50_ values of 25 μM. For highly active compounds where dilution failed to titrate away drug activity (i.e., computed IC_50_ ≤ 0.001 μM), we assigned IC_50_ values of 0.001 μM.

### Cell culture and reagents

U2OS, B16-F10, or C33A cells purchased from the American Type Culture Collection were cultured in Dulbecco’s modified Eagle’s medium (Gibco, Life Technologies, NY, USA) supplemented with 10% fetal bovine serum (Sigma-Aldrich, MO, USA) and 1% penicillin-streptomycin (Gibco, Life Technologies, NY, USA) at 37°C under 5% CO_2_. The cells were transfected with siRNAs using Lipofectamine RNAiMAX (Invitrogen) and DNA plasmids using FuGENE HD reagents (Promega, Madison, WI, USA).

### Antibodies and immunoblotting

Immunoblot analysis for U2OS cells was performed as described (*61*) using the following antibodies: anti-BMAL1 (14020, Cell Signaling, Danvers, MA, USA), anti-CLOCK (sc-6927, Santa Cruz Biotechnology, Dallas, TX, USA), anti-PER2 (20359-1-AP, Proteintech, Chicago, IL, USA), CRY1 (A302-614A, Bethyl Laboratories, Montgomery, TX, USA), anti-CRY2 (13997-1-AP, Proteintech), anti–REV-ERBα (13418, Cell Signaling), anti–REV-ERBβ (GTX115322, GeneTex, Irvine, CA, USA), anti-CCNB1 (#4138, Cell Signaling), anti-CCND1 (ab134175, Abcam, Cambridge, MA, USA), anti-CDK4 (12790, Cell Signaling), anti-AKT (4685S, Cell Signaling), anti-phospho AKT Ser^473^ (pAKT) (4060S, Cell Signaling), anti-p44/42 mitogen-activated protein kinase (MAPK) (Erk1/2) (4695S, Cell Signaling), anti–phospho-p44/42 MAPK (Erk1/2)-Thr^202^/Tyr^204^ (pERK) (4370S, Cell Signaling), anti–caspase-3 (9662S, Cell Signaling), anti-HSF1 (sc-17756, Santa Cruz Biotechnology), anti-HSP70 (4872S, Cell Signaling), anti-HSP90AA1 (8165S, Cell Signaling), anti-HSP90AB (7411S, Cell Signaling), anti-HSP90B1 (Grp94) (2104S, Cell Signaling), and anti-TRAP1 antibody (GTX102017, GeneTex). Anti-GAPDH (glyceraldehyde-3-phosphate dehydrogenase) (sc25778, Santa Cruz Biotechnology), anti-tubulin (ab18251, Abcam), and anti–β-actin (4967S, Cell Signaling) were used as loading control antibodies.

### Immunoprecipitation

For immunoprecipitation analysis (fig. S9B), the U2OS cell lysates were harvested in radioimmunoprecipitation assay buffer (9806, Cell Signaling) with protease inhibitor cocktail (11697498001, Sigma-Aldrich, St. Louis, MO, USA). Protein A–coated magnetic beads (10006D, Thermo Fisher Scientific, Waltham, MA, USA) were preincubated with 2 μg of CRY1 (A302-614A, Bethyl Laboratories), CRY2 (A302-615A, Bethyl Laboratories), and normal rabbit immunoglobulin G (2729, Cell Signaling) at 4°C for 6 hours. The antibody-conjugated beads were incubated with lysate containing equal amounts of total protein at 4°C overnight. The final immune complexes were analyzed by immunoblot assay using anti-HSP90AA1 (HSP90 α) (SMC-108D, StressMarq Biosciences, Victoria BC V8N 4G0, Canada), anti-HSP90AB1 (HSP90 β) (37-9400, Thermo Fisher Scientific), CRY1 (A302-614A, Bethyl Laboratories), CRY2 (A302-615A, Bethyl Laboratories), and anti-actin (4967S, Cell Signaling).

### Transfection of siRNA

siRNAs targeting human HSP90AA1 (SI00075971), HSP90AB1 (SI02780561), HSP90B1 (SI02663738), and TRAP1 (SI00115150) were purchased from Qiagen. Transfection of 100 pmol of siRNAs per well in U2OS cells was conducted with Lipofectamine RNAiMAX Transfection Reagent (13778075, Invitrogen) according to the manufacturer’s instructions. Immunoblotting was performed 48 hours after transfection as described above.

### Circadian rhythm reporter cell line generation

p*Per2*-dLuc lentiviral reporter was provided by A. C. Liu at the Department of Physiology and Functional Genomics, University of Florida. C33A or B16-F10 cell reporter lines stably expressing p*Per2*-dLuc were generated according to stable transduction protocol using lentivirus-mediated gene delivery (*62*).

### CRISPR gene knockout cell line generation

For generation of control and *Bmal1* knockout B16-F10 cell lines (CTL-B16 and *Bmal1*-KO B16), we purchased the scrambled single-guide RNA (sgRNA) control (pCRISPR-CG12) and sgRNA/Cas9 all-in-one expression clone targeting Arntl (MCP276671-CG12-3-10) from GeneCopoeia (Rockville, MD, USA) and followed the manufacturer’s CRISPR genome editing protocol. The cells were grown with the addition of G418 sulfate selection marker (S3028, Selleckchem, Pittsburgh, PA, USA) for 2 weeks before single cells were sorted using fluorescence-activated cell sorting (FACS Melody, BD). After selection, Western blot and chrono-pharmacological experiments were performed as described. BMAL1, REV-ERBα (NR1D1), and REV-ERBβ (NR1D2) were silenced in U2OS *Arntl::*dLUC cells through CRISPR editing using all-in-one (Cas9 nuclease + sgRNA) plasmids acquired from GeneCopoeia. U2OS cells were seeded at a density of 1.3 × 10^5^ cells per well. On the day of transfection, cells are optimal at 70 to 90% confluency. Cells were transfected with Lipofectamine 3000 according to the manufacturers’ instructions. The following day, mCherry-positive cells were sorted by FACS as single cells into 96-well plates. Resulting clonal lines were then screened by immunoblot for silencing of target. Because clones derived from the parental U2OS Arntl::dLUC line exhibit heterogeneity in intensity of luciferase expression (regardless of transfection), edited cell lines were screened for luciferase expression. BMAL1 gRNA (GGTTCATGGTACCTTCCATG, GeneCopoeia catalog no. HCP271154-CG01-3-B-b, mCherry), REV-ERBα gRNA (TGGTGAAGACATGACGACCC, GeneCopoeia catalog no. HCP222925-CG01-3-10-a, mCherry), and REV-ERBβ gRNA (CAAGACCCGGCTCGCTCCTT, GeneCopoeia catalog no. HCP222925-CG01-3-B-b, mCherry).

### Bioluminescence recording and data analysis

A total of 1.5 × 10^5^ p*Per2* or p*Bmal1* reporter cells were seeded per 35-mm dish. After the 100 nM dex pulse (~30 min) (D2915, Sigma-Aldrich), real-time bioluminescence of the cells was monitored using a LumiCycle luminometer (Actimetrics, Wilmette, IL, USA), and period and amplitude of the luminescence data were determined through the LumiCycle software program (Actimetrics). For real-time bioluminescence recordings in 96-well plates, 5 × 10^3^ clock reporter cells were seeded in 96-well plates and monitored using a Cytation 5 multimode reader (BioTek) after the 100 nM dex pulse (~30 min) under various experimental condition as described in the figure legends (Fig. 4, D to G). The following period and amplitude analysis of the luminescence data was performed using Biodare2 rhythm analysis program (https://biodare2.ed.ac.uk/).

### Cell proliferation assay

A total of 5 × 10^3^ U2OS or B16-F10 cells were seeded per well in 96-well plates. After 1-hour dex synchronization (100 nM), the cells were treated with vehicle or candidate anticancer drugs (0.01 to 1 μM) at 6-hour intervals over the course of 24 hours, and the subsequent time course of cell viability was determined colorimetrically 48 hours later with Alamar Blue reagent (DAL1025, Thermo Fisher Scientific) (Fig. 2A). Briefly, before addition of the Alamar Blue reagent, microplates were subjected to removal of media by aspiration followed by addition of a mixture of 100 μl of fresh media and 10 μl of Alamar Blue reagent. The plates were incubated for 6 hours at 37°C, and fluorescence activity was monitored at 555-nm excitation wavelength and 595-nm emission wavelength on the Epoch Microplate Spectrophotometer.

### FUCCI reporter cell line generation and time-lapse cell cycle analysis

For the FUCCI-based live-cell imaging, we purchased a plasmid (pBOB-EF1-FastFUCCI-Puro) encoding fluorescent probes [mKO2-hCDT1 for cell growth (G_0_-G_1_) and mAG-hGeminin for DNA replication (S) and cell division (G_2_-M) phases] from Addgene (86849). After the cell cycle reporter was stably introduced into control or BMAL1 knockout U2OS cells according to the previous protocol (*29*), the FUCCI reporter cells were seeded in a 384-well plate and synchronized with 100 nM dex for 1 hour. Twenty-four hours later, phases of the cell cycle were monitored for 48 or 72 hours with the Cytation 5 cell imaging multimode reader (BioTek, Winooski, VT, USA) using GFP (excitation, 492 nm and emission, 514 nm) and RFP (excitation, 579 nm and emission, 604 nm) filter sets with the same light exposure time. Real-time cell counting analysis was performed using BioTek Gen5 data analysis software to measure cells positive for FUCCI sensor (RFP/GFP) in the reporter cells.

### RNA extraction and reverse transcription and quantitative PCR

Total RNA was isolated from cell (U2OS and B6-F10) or mouse tissue (melanoma tumor, liver) samples using the RNeasy Plus Mini Kit (74134, Qiagen) according to the manufacturer’s protocol. Equal amounts of complementary DNA were synthesized using the Invitrogen Superscript II RT First-Strand Synthesis System according to the manufacturer’s protocol using 2 μl of random hexamers and 9 μl of RNA (Life Technologies, Carlsbad, CA, USA). qPCR was performed using the SYBR Green PCR Master Mix (Applied Biosystems) and 10 μM of the forward and reverse primers. All qPCR reactions were conducted at 50°C for 2 min, 95°C for 10 min, and then 40 cycles of 95°C for 15 s and 60°C for 1 min. The specificity of the reaction was assessed by melt curve analysis. The relative gene expression of each sample was quantified using the comparative Ct method. Samples were normalized to GAPDH, which was used as an endogenous control for all experiments. Experiments were performed using a ViiA7 Real-Time PCR machine (Thermo Fisher Scientific).

### Animal studies

C57BL/6J mice (Jackson laboratories, Bar Harbor, ME, USA) were housed (≤5 per cage) under 12-hour light:12-hour dark conditions with food and water available ad libitum. Male mice (2 to 3 months old) were used in all experiments. After acclimation with standard lighting conditions of 12-hour light:12-hour dark, with lights on from 5:00 a.m. (ZT0) to 5:00 p.m. (ZT12) for 2 weeks, the mice were injected with 1 × 10^6^ B16-F10 cells subcutaneously along the right flank. For CRISPR control B16-F10 (CTL-B16) and *Bmal1* knockout (*Bmal1* KO-B16) tumors, mice were briefly anesthetized with isoflurane, and 100-μl volume of cells was injected subcutaneously along the right (WT-B16) and left (*Bmal1*-B16) flanks. After 7 to 10 days of the injection, the mice were orally administered daily with vehicle (distilled water) or 17-DMAG at ZT3 or ZT15. Locomotor activity was monitored as described (*63*). Tumor growth was measured with a digital caliper. Tumor weight was computed as follows: (length × width^2^) / 2. In all animal experiments, mice were sacrificed when the tumor exceeded 10 mm in mean diameter (approximately a volume at 1500 mm^3^). Tumor and liver tissues were collected at ZT3 and ZT15 to measure time-of-day changes in target gene transcripts or proteins with qPCR or Western blot analysis as described.

### In vitro and in vivo chronopharmacology

For pharmacological inhibition of HSP90 activity in U2OS or B16-F10 cells (Figs. 2 and 4 and figs. S5 and S8), the following drugs were used in various experimental settings as described (Fig. 2A): 17-AAG (A10010-10, AdooQ Bioscience, Irvine, CA, USA), 17-DMAG (A10011, AdooQ Bioscience), AZD4547 (S2801, Selleckchem, Pittsburgh, PA, USA), MK1775 (S1525, Selleckchem), triptolide (S3604, Selleckchem), dinaciclib (S2768, Selleckchem), NMS-873 (A12377-5, AdooQ Bioscience), raltitrexed (A10776-10, AdooQ Bioscience), and FK866 (A11279-10, AdooQ Bioscience). For in vivo animal studies, 17-DMAG HCl (S1142, Selleckchem) was dissolved in water and administered by oral gavage daily at 10 to 20 mg/kg at ZT3 and ZT15 after measurable tumors were formed following B16 melanoma cell injection as described above. For evaluation of antitumor activity of the drug, tumor growth was measured with a digital caliper daily during drug administration. Additional details for each experiment are given in Fig. 5 legend. For chrono-pharmacokinetic analysis, the blood samples (3×) of mice entrained at ZT3 or ZT15 as described above were collected with anticoagulant (K_2_EDTA)–coated BD Vacutainer tubes (02-657-32, Thermo Fisher Scientific) from the eyes at 5, 15, 30, 45, 60, 120, 240, 420, and 1140 min after oral dose of vehicle or 17-DMAG (75 mg/kg). After centrifugation at 4°C at 1800*g* for 10 min, plasma fractions were separated from the blood samples and were carefully transferred to 1.5-ml Eppendorf tubes and snap-frozen in dry ice before high-performance liquid chromatography and pharmacokinetic analysis performed by NorthEast BioLab (Hamden, CT, USA).

### Statistical analysis

All statistical tests used in this study were completed with Prism7 GraphPad software. For making multiple comparisons, we used one-way or two-way analysis of variance (ANOVA) followed by Bonferroni’s and Tukey’s multiple comparisons test. For comparing the average of two means, we used Student’s *t* test (two-tailed paired or unpaired) to reject the null hypothesis (*P* < 0.05).

## SUPPLEMENTARY MATERIALS

Supplementary material for this article is available at http://advances.sciencemag.org/cgi/content/full/7/7/eabd2645/DC1

## Appendix

View/request a protocol for this paper from *Bio-protocol*.
